# Supplementary material for: Nonlinear topological symmetry protection in a dissipative system
Source: Nat Commun. 2024 Feb 15;15:1398. doi: 10.1038/s41467-023-44640-x (PMC10869785; doi:10.1038/s41467-023-44640-x)
Supplement: Supplementary file 1 — Supplementary information [file 41467_2023_44640_MOESM1_ESM.pdf]

Supplementary information – Nonlinear topological symmetry protection in a dissipative system

Stéphane Coen,[1,2,*] Bruno Garbin,[1,2,†] Gang Xu,[1,2,‡] Liam Quinn,[1,2] Nathan Goldman,[3] Gian-Luca Oppo,[4] Miro Erkintalo,[1,2] Stuart G. Murdoch,[1,2] and Julien Fatome[1,2,5]

[1] *Physics Department, The University of Auckland, Private Bag 92019, Auckland 1142, New Zealand*
[2] *The Dodd-Walls Centre for Photonic and Quantum Technologies, Dunedin, New Zealand*
[3] *Center for Nonlinear Phenomena and Complex Systems, Université Libre de Bruxelles, CP 231, B-1050 Brussels, Belgium*
[4] *SUPA and Department of Physics, University of Strathclyde, Glasgow G4 0NG, Scotland, European Union*
[5] *Laboratoire Interdisciplinaire Carnot de Bourgogne (ICB), UMR 6303 CNRS, Université de Bourgogne, 9 Avenue Alain Savary, BP 47870, F-21078 Dijon, France*

This article contains supplementary information to the manuscript entitled "*Nonlinear topological symmetry protection in a dissipative system.*" In particular, we present results from additional simulations, theoretical analyses, and experiments that further elucidate the physics and robustness of the alternating P2 spontaneous symmetry breaking phenomenon reported in our study.

I. NUMERICAL SIMULATION OF P2 SSB

To confirm that the conclusions drawn from the mean-field description, Eqs. (13)–(14) of the main Article, are correct, we present here numerical simulation results of P2 spontaneous symmetry breaking (SSB) using a full lumped iterative map model of our optical fiber resonator. Specifically, we proceed by integrating the classical equation of motion for the x and y modes [Eq. (3) of the main Article and a similar equation for ψ_x, with the addition of kinetic terms] over one resonator roundtrip time t_R, followed by application of the resonator boundary conditions, Eq. (15) of the main Article, and the process is repeated over many roundtrips until steady state field distributions are obtained.

Using this approach, we have reproduced numerically the experimental observation of P2 SSB reported in Figs. 3a–c of the main Article. We used the same normalized cavity detuning $\Delta = 10$ and normalized peak driving power $X = 50$, in a synchronous nanosecond pulse driving configuration, as in the experiments. We set $A = 1$, $B = 2/3$ and $C = 1/3$, which correspond to modes with linear states of polarizations. Finally, we introduced a deviation from an exact π-phase defect between the modes of the resonator by setting $\delta_\pi/\alpha = 2$ to illustrate the robustness of the system against such imperfection. The results of the simulations are presented in Fig. S1. Panels a–b show the real part of the amplitudes of the x and y modes, ψ_x and ψ_y, across the driving pulses over two consecutive roundtrips. We can first observe that the y-mode is quite significant in the conditions of the simulation, despite being undriven. This confirms the role of parametric generation. Second, the y mode clearly flips its sign, $\psi_y \rightleftharpoons -\psi_y$, over subsequent roundtrips while the x mode amplitude keeps stationary. The same behavior is observed for the imaginary part of the field (not shown), and this clearly highlights that the system is attracted to a y-mode roundtrip-to-roundtrip phase step of π, irrespective of the non-zero value of δ_π. Panel c shows the corresponding hybrid mode intensity profiles, $|\psi_+|^2$ and $|\psi_-|^2$. Their evolution over 20 roundtrips is then shown in panels d and e, with the total intensity in panel f, using the same format as in Figs. 3a–c of the main Article. The alternation of the + and − hybrid modes, $\psi_+ \rightleftharpoons \psi_-$, over subsequent roundtrips, which is associated with the sign flip of the y mode, is clearly visible. At the same time, the total intensity appears constant [Fig. S1f]. Overall,

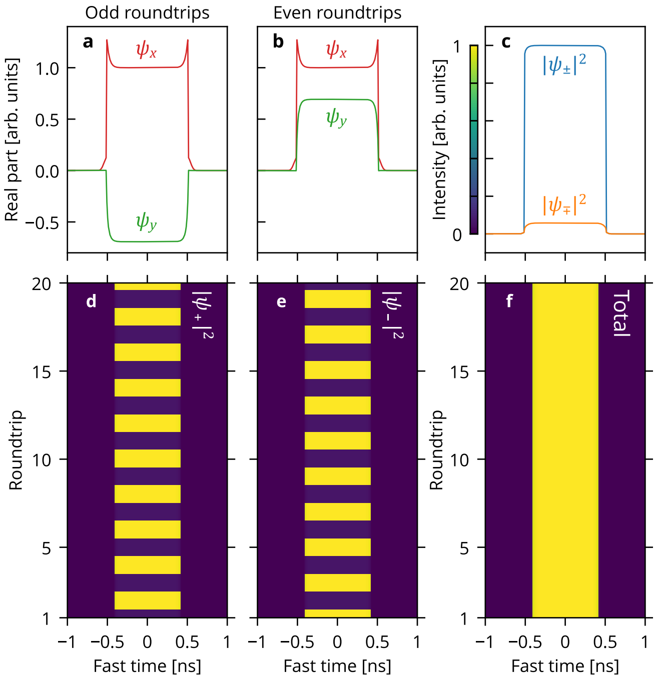

Figure S1 | Numerical simulations of P2 SSB. a, b Real part of the stationary amplitudes of the x and y modes ψ_x and ψ_y over even- and odd-roundtrips when driving the x mode of the resonator ($\chi = 0$). **c** Corresponding hybrid mode intensities $|\psi_+|^2$ and $|\psi_-|^2$, with (**d**–**f**) their roundtrip-to-roundtrip evolution, together with that of the total intensity, as indicated. All results obtained by simulation of an iterative map model of the resonator with $X = 50$, $\Delta = 10$, and $\delta_\pi/\alpha = 2$. Other parameters given in the text.

---

* Corresponding author, s.coen@auckland.ac.nz
† Present address: NcodiN SAS, 10 Boulevard Thomas Gobert, F-91120 Palaiseau, France
‡ Present address: School of Optical and Electronic Information, Huazhong University of Science and Technology, 1037 Luoyu Road, Wuhan, China

our simulations are in excellent agreement with the experimental observations of Fig. 3 of the main Article.

II. RANGE OF EXISTENCE OF P2 SSB

Figure S2 illustrates the range of existence of stable homogenous P2 SSB solutions in the (δ_π, X) parameter space, calculated numerically in the mean-field framework for $A = 1$, $B = 2/3$, $C = 1/3$. In that plot, color represents the span of normalized detuning over which P2 SSB exists for particular parameters. That span is calculated from the threshold of SSB to its disappearance, or to the smallest detuning value above threshold for which P2 SSB solutions become unstable, whichever arises first. Not surprisingly, the detuning span widens (lighter color) with larger driving power, associated with a larger nonlinear tilt of the resonances. More interestingly, we find that P2 SSB is quite tolerant with respect to a deviation from an exact π-phase-shift defect. This is especially the case for positive values of δ_π, corresponding to the y-mode resonance shifted in the same direction as the nonlinear tilt of the x-mode resonance. Indeed it is under that condition that the x and y components of the field can simultaneously be closer to their respective resonance.

Detailed analysis of Eqs. (19)–(20) of the main Article provides analytical expressions for the boundaries of the existence range of P2 SSB. We find that there is an overall driving power threshold, $X \geq 1/C$, and approximate boundaries for δ_π given by

$$\frac{A-B}{C} - \sqrt{CX-1} \lesssim \frac{\delta_\pi}{\alpha} \lesssim (A-B+C)X\,. \qquad \text{(S1)}$$

For $A = 1$, $B = 2/3$, $C = 1/3$, we have $X \geq 3$ and

$$1 - \sqrt{X/3-1} \lesssim \frac{\delta_\pi}{\alpha} \lesssim 2X/3\,. \qquad \text{(S2)}$$

These boundaries are shown as the red dashed curves in Fig. S2 and found to match well with the numerical predictions.

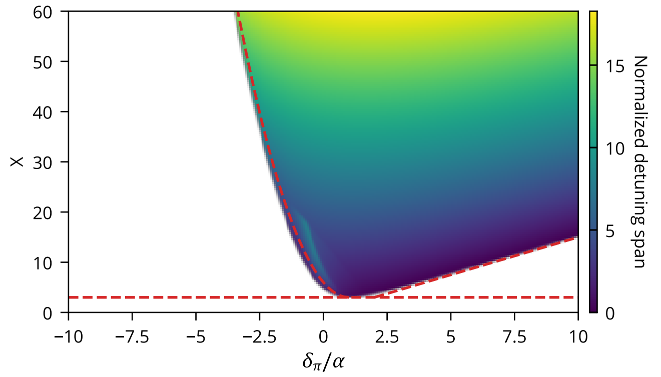

Figure S2 | Range of existence of P2 SSB. The color plot represents numerical predictions of the span of normalized detuning over which P2 SSB is stable, calculated from threshold, as a function of driving power X and deviation from a perfect π-phase defect, δ_π. White denotes the absence of P2 SSB solutions. $A = 1$, $B = 2/3$, $C = 1/3$. Red dashed lines are analytical predictions for the boundaries of the existence range.

III. RESONANCE SCAN

To complete the data presented in Fig. 3 of the main Article, we show in Fig. S3 measurements of the intensities of (a) the x and y modes taken in parallel with (b) those of the + and − hybrid modes for a range of detunings across the resonance. This plot constitutes an experimental counterpart of the conceptual diagram presented in Fig. 1c of the main Article and clearly illustrates how SSB of the hybrid modes is associated with parametric generation of the y mode (both highlighted by the yellow band). Note that the thresholds do not fully match between panels a and b due to the effect of the detection noise floor on the y-mode intensity $|\psi_y|^2$.

For these measurements, we used a 57 m long normal dispersion resonator, with a roundtrip time $t_R = 274$ ns, and a resonance linewidth $\Delta\omega/(2\pi) = 104$ kHz. The data were obtained by sampling the peak powers of the output pulses over subsequent roundtrips as the detuning was ramped up.

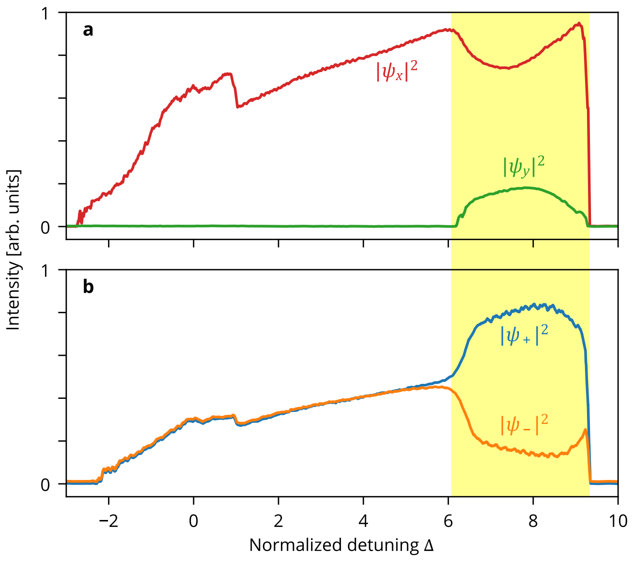

Figure S3 | Resonance scan. Intensities of (**a**) the x and y modes and (**b**) the + and − modes versus cavity detuning recorded in parallel in the same conditions. The yellow band highlights how parametric generation of the y mode is associated with SSB of the hybrid modes. The normalized driving power was set to $X \sim 15$.

IV. ADDITIONAL RANDOMNESS TESTS

To further evaluate the robustness of our system against imperfections, we have tested the randomness of the P2 SSB state selection process as a function of δ_π, i.e., in presence of deviations of the y-mode phase defect from an exact value of π. Different values of δ_π were obtained by manipulating the fiber polarization controller FPC_π placed inside the resonator (see Fig. 2 of the main Article). As this procedure affects the state of polarization of the modes of the resonator, FPC_{in} was then adjusted accordingly each time to ensure that we keep driving only the x-mode. For each value of δ_π, we collected sequences of 320,000 state selection events using the procedure de-

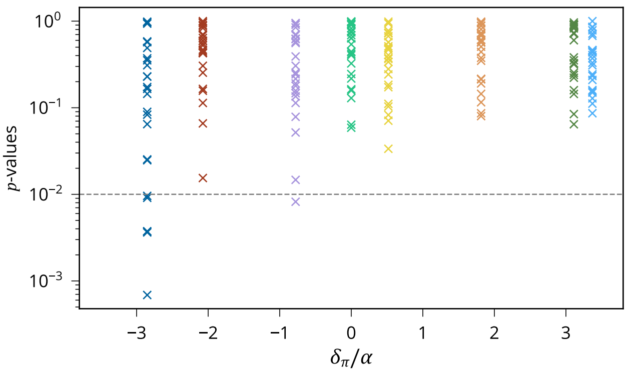

Figure S4 | Randomness test results versus δ_π. p-values of the monobit test for various deviations from an exact π-phase defect. For each value of δ_π (grouped by color), the tests are conducted over 320,000 events partitioned into 25 sub-sequences.

scribed in the main Article. These were each partitioned into 25 sub-sequences, for which we generated p-values for the monobit test (whether the two states appear with the same likelihood, as expected for a truly random process). Those p-values are plotted in Fig. S4. As can be seen, the large majority of p-values are clustered above the 0.01 significance level, indicating randomness. Only for δ_π around -3 do we see evidence of non-randomness, with a significant proportion (more than 4 %) of p-values appearing too low. This can be attributed to the system being too close to the threshold of SSB in those conditions, such that we cannot reliably distinguish the two states. Note that these data have been obtained with the same resonator as that used for Fig. S3.

Overall, the measurements presented in Fig. S4 confirm that the randomness of the state selection process is robust against deviations from an exact π-phase defect, and support our hypothesis that the Möbius topology of the resonator is protected by the nonlinearity.

V. OPTICAL SPECTRUM OF P2 BRIGHT SYMMETRY-BROKEN CAVITY SOLITONS

We have evaluated the temporal duration of the P2 bright symmetry-broken cavity solitons reported in Figs. 5d–f of the main Article by measuring the optical intensity spectra of the two hybrid modes. These measurements are reported in Figure S5 (blue and orange curves). By fitting these spectra with a hyperbolic-secant squared profile (black dashed curve), we estimate the temporal duration of the pulses to be 1.6 ps (full-width at half maximum).

VI. LONG-TERM OBSERVATION OF P2 LOCALIZED STRUCTURES

To assess the robustness of P2 localized structures, we have performed additional observations over very large number of roundtrips. In Fig. S6, we first report such measurements for a single P2 domain wall obtained in the same resonator and in the same conditions ($X = 50$, $\Delta = 10$) as that used for the results of Figs. 5a–c of

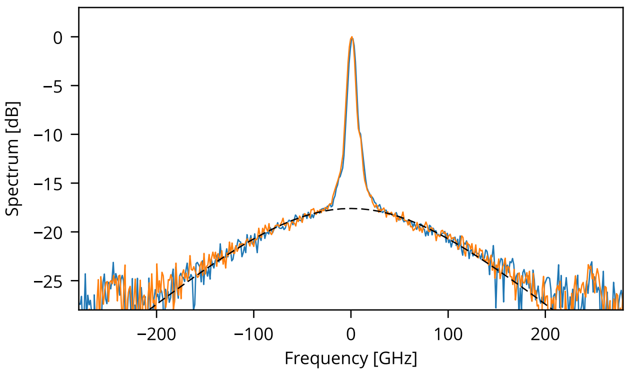

Figure S5 | Spectra of P2 bright symmetry-broken cavity solitons. Blue and orange curves correspond to the two hybrid modes while the black dashed line is a numerical fit to a sech^2 spectrum, consistent with a temporal duration of 1.6 ps. The measurements were performed with $X = 30$ and $\Delta = 14.5$ (same as in Figs. 5d–f of the main Article).

the main Article. Temporal intensity profiles of the two hybrid modes were taken every 30 seconds over more than 30 minutes (corresponding to more than 30 billion roundtrips in the resonator) and are presented in a 3D color plot. For the sake of clarity, only odd roundtrips are plotted to conceal the alternating dynamic. These data have been obtained without taking any specific precautions, yet do not reveal any appreciable changes in the domain wall characteristics over the recording period. In comparison, dissipative polarization domain walls unprotected by the alternating Möbius topology have only been reported over 30 seconds [31]. Our results therefore represent a major improvement and a major step forward for potential practical applications of polarization domain walls.

Next, using a similar format as in Fig. S6, we present in Fig. S7 long-term observations of a P2 symmetry-broken bright cavity soliton. Here we have plotted separately the

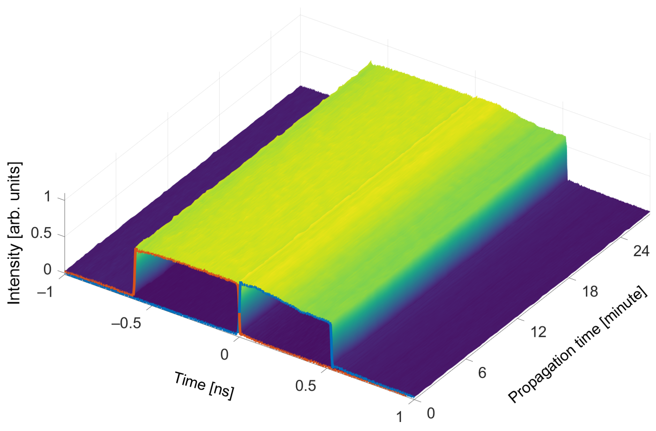

Figure S6 | Long-term observation of a P2 domain wall. The 3D graph is made of a succession of temporal intensity profiles of the + and − hybrid modes measured every 30 seconds and plotted as a function of propagation time in the resonator. For clarity, only odd roundtrips are shown to conceal the alternating dynamics. The orange and blue curves correspond to the profiles recorded at the first roundtrip. $X = 50$ and $\Delta = 10$.

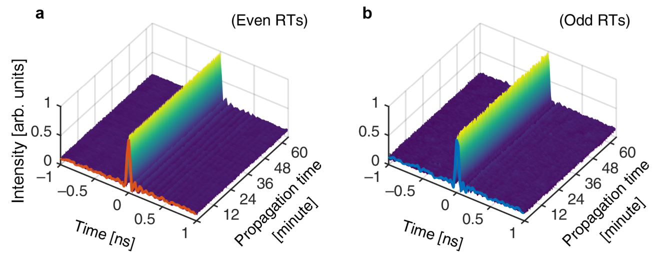

Figure S7 | Long-term observation of a P2 bright symmetry-broken cavity soliton. Using a similar format as in Fig. S6, the two plots represent the evolution of the temporal intensity profile of (**a**) the + hybrid mode at even roundtrips and (**b**) the − hybrid mode at odd roundtrips with only a single soliton in the resonator. $X = 10$ and $\Delta = 5.5$.

evolution of the intensity profile of (a) the + hybrid mode at even roundtrips and (b) the − hybrid mode at odd roundtrips. The two graphs are identical as expected, with no perceivable changes over one hour, which again confirms the robustness of our system. These data were taken in the same anomalous dispersion resonator as that described in our main Article, using the same driving power ($X = 10$) as that used for the P2 breather reported in Figs. 5g–k but with a slightly higher detuning ($\Delta = 5.5$) to avoid the breathing dynamics [76].
